# Measuring the Impact of Healthcare Provider Availability on Quality of Care and Disease Burden in Ohio: A Cross-Sectional Study

**DOI:** 10.1007/s11606-024-09312-6

**Published:** 2025-01-07

**Authors:** Samuel Borgemenke, D’Nair Newsom, Halie Leftwich, Lucille Gideon, Elizabeth A. Beverly

**Affiliations:** 1 Department of Medicine, 1 Ohio University, Ohio University Heritage College of Osteopathic Medicine, Athens, USA; 2 Department of Primary Care, The Ohio University Diabetes Institute, 1 Ohio University, Athens, OH USA

**Keywords:** healthcare, quality of care, disease burden, appalachia, Ohio, providers

## Abstract

**Background:**

Chronic lower respiratory disease, heart disease, and diabetes have a higher prevalence in rural areas. Previous studies raise concerns that a lower supply of physicians is associated with negative outcomes.

**Objective:**

To assess disease burden across the 88 counties in Ohio, including Appalachian and non-Appalachian counties, and examine associations with the number of healthcare providers.

**Methods:**

We utilized data sourced from the Centers for Medicare & Medicaid Services (CMS) and the Mapping Medicare Disparities tool. We conducted ANOVA to compare the mean number of primary care physicians (PCP), specialty physicians, advanced practice providers (APP), and other healthcare providers for Ohio counties. We calculated mean prevalence, principal cost, and prevention quality indicator (PQI) by health condition. We analyzed the relationship between healthcare providers and the PQI across counties, and examined differences in healthcare providers and disease burden between Appalachian and non-Appalachian regions.

**Results:**

The mean number of providers per 100,000 people significantly differed between PCP, specialty physicians, APP, and other healthcare providers (*F* = 13.9, *P* < 0.001). The prevalence of hypertension (mean = 67.0, SD = 2.2) and diabetes (mean = 27.1, SD = 2.4) was the highest of the selected conditions. The number of preventable hospitalizations from chronic conditions (mean = 2024.9, SD = 526.8) was significantly higher (*P* < 0.001) than the number of preventable hospitalizations from acute conditions (mean = 851.6, SD = 262.2). The multivariate mixed effects model of PCP*Specialist*APP*Other was the best predictive model for all health conditions (*P* < 0.001). COPD (mean = 17.4, SD = 2.8) and diabetes (mean = 28.4, SD = 2.3) in Appalachian counties were significantly higher (*P* < 0.001) than COPD (mean = 13.7, SD = 1.9) and diabetes (mean = 26.2, SD = 2.1) in non-Appalachian counties.

**Conclusion:**

This study found higher PQI scores for chronic conditions than acute conditions, indicating the need for higher-quality outpatient care to prevent avoidable hospital admissions. Further, Appalachian counties had fewer other healthcare providers and many counties lacked specialist physicians, highlighting significant disparities in healthcare access in Appalachian Ohio.

## BACKGROUND

Preventable hospitalizations are hospital stays for acute and chronic conditions that could have been avoided with appropriate primary care services.^1^ High rates of preventable hospitalizations represent emergency department and urgent care overuse as well as indicate issues with accessibility and quality of outpatien primary care services.^1^ In 2017, preventable hospitalizations cost approximately $33.7 billion in the United States (U.S.).^1^ Certain populations are more likely to experience preventable hospitalizations,^1,2^ particularly those historically affected by inequities due to race, ethnicity, socioeconomic status (SES), age, geographic location, and other structural factors linked to discrimination or exclusion.^2,3^

A CDC study conducted from 2010 to 2017 found nonmetropolitan counties had higher percentages of potentially excess deaths from unintentional injury, stroke, chronic lower respiratory disease (CLRD), heart disease, and cancer.^4^ In 1999, the mortality rate in rural areas was 7% higher than that in urban areas, and by 2019, this disparity rose to 20%.^3^ The most recent data from the Ohio State Health Assessment identified COVID-19 as one of the top five causes of increasing mortality rate.^5^ This assessment also revealed that COVID-19, as well as the general mortality rate, was higher in Ohio compared to the rest of the U.S.^5^ However, this study did not address questions about the correlation between physician density and their statewide distribution, and its impact on health equity.^5^

Recently, health policies have been developed to help reduce the number of hospitalizations for conditions that could be managed through ambulatory care, signaling a call to action for improved primary and preventive care. Billions of dollars have already been spent to address this including the expansion of Medicaid to people with household incomes at or below 138% of the federal poverty level.^6^ The passing of the Affordable Care Act saw reductions in preventable hospitalization visits in many acute and chronic conditions.^1,6^ Further, inpatient days for adults decreased, while discharge rates increased, and overall costs for hospitals decreased for ambulatory care-sensitive conditions.^1,6^

The healthcare system relies on primary care physicians as the first point of contact for individuals seeking medical care. Their responsibilities include promoting health, preventing and managing diseases, and coordinating comprehensive patient care.^7^ However, the U.S. faces a shortage of primary care providers, with an estimated 17,000 primary care providers needed to meet healthcare demands.^7,8^ Rural areas, like Appalachian Ohio, are experiencing workforce shortage, which negatively impacts the health of the population.^8,9^ For example, 26 of Ohio’s 32 Appalachian counties are considered rural.^10^ Moreover, 28 of the 32 Appalachian counties are designated health professional shortage areas.^11^ Appalachian Ohio has 30% fewer primary care physicians compared to non-Appalachian Ohio, 41% fewer mental health providers, 65% fewer specialty physicians, and 33% fewer dentists.^12^ Studies have also shown these communities rely heavily on government-assisted aid programs, struggle with internet bandwidth for telehealth visits, and face transportation difficulties that result in fewer healthcare check-ins and more emergency department visits.^7–9^ Prior research raises concerns that a lower supply of physicians is associated with higher mortality,^13^ underscoring the need to enhance access to care.^13–15^

Geographical differences within the U.S. contribute to some regions having a higher density of rural areas than metropolitan areas.^16^ The South and Midwest regions have higher percentages of the population living in rural areas, with Ohio being one of the top five states or territories with the largest rural population.^16^ These rural regions face gaps in care, resources, and accessibility that are not fully understood. More research is needed to understand these gaps, particularly in areas endemic to health inequities. Research on the burden of disease and quality of care in both acute and chronic conditions in Ohio may identify new approaches to reduce disparities unique to rural and underserved regions.

The primary objective of this study was to assess disease burden across the 88 counties in Ohio, including Appalachian and non-Appalachian counties, and examine associations with the number of healthcare providers serving in these areas.

## METHODS

### Ethics Approval

This research did not qualify as human subjects research under federal regulations because it was de-identified, publicly available data. Thus, it was exempt from Institutional Review Board (IRB) review. We followed the Strengthening the Reporting of Observational Studies in Epidemiology (STROBE) guidelines.^17^

### Data Source

We sourced data from the Centers for Medicare & Medicaid Services (CMS)^18,19^ through their healthcare provider database and Mapping Medicare Disparities tool.

### Inclusion Criteria

The study required data from across Ohio and classified counties as Appalachian and non-Appalachian to include participants and conditions.

### Variables

Selected variables included the prevalence of healthcare providers (broken down by primary care physicians, specialty physicians, advanced practice providers, and other healthcare providers) and health conditions, as well as the quality of care for those health conditions. Variables sourced from CMS provided statistics from all 88 counties in Ohio. The complete list of the defined variables is as follows:

#### Primary Care Physicians (PCP)

Doctors of medicine (MD) and doctors of osteopathy (DO) providing direct patient care who practice principally in primary care specialties—general or family practice, geriatrics, general internal medicine, pediatrics, and obstetrics and gynecology from 2022.^19,20^

#### Specialty Physicians

Doctors of medicine (MD) and doctors of osteopathy (DO) providing direct patient care who practice principally in specialties not otherwise defined as primary care specialties, such as surgeons, surgical subspecialists, anesthesiologists, and medicine-based subspecialists, from 2022.^19^

#### Advanced Practice Providers (APP)

Healthcare providers who identified as certified registered nurse anesthetists, physician assistants, nurse practitioners, clinical nurse specialists, or anesthesia assistant, from 2022.^19,21^

#### Other Healthcare Providers

Healthcare providers who identified as podiatrists, optometrists, physical therapists, chiropractors, registered dieticians, social workers, audiologists, clinical psychologists, occupational therapists, speech pathologist, dentist, or medical geneticist from 2022.

#### Prevalence of Health Conditions

The population-adjusted number of cases for a given disease (i.e., asthma, acute myocardial infarction, hypertension, COPD, diabetes) from 2022.^18^ We selected these specific diseases because of existing literature showing increased prevalence of cardiovascular disease and diabetes in Ohio, especially in the Appalachian region.^22–24^

#### Principle Cost

The average price per Medicare beneficiary for various conditions in 2022.^18^

#### Prevention Quality Indicator (PQI)

The total number of hospitalizations for a given condition per 100,000 persons by county for 2022.^25^

#### Preventable Hospital Stays

The number of hospital stays for ambulatory care-sensitive conditions per 100,000 Medicare enrollees (diabetes with short- or long-term complications, uncontrolled diabetes without complications, diabetes with lower-extremity amputation, chronic obstructive pulmonary disease, asthma, hypertension, heart failure, dehydration, bacterial pneumonia, or urinary tract infection) in 2022.^18^ Ambulatory care-sensitive conditions are defined as conditions where outpatient care and/or early intervention could potentially prevent hospitalization.^25^

#### Diabetes Short-Term Complications

Hospital admissions for a primary diagnosis of diabetes accompanied by short-term complications (such as ketoacidosis, hyperosmolarity, or coma) per 100,000 individuals aged 18 and above in 2022. This excludes admissions related to obstetrics and transfers from external institutions.^26^

#### Diabetes Long-Term Complications

Hospital admissions for a primary diagnosis of diabetes accompanied by long-term complications (ICD-9-CM diagnosis of renal, eye, neurological, circulatory, or complications not otherwise specified) per 100,000 individuals aged 18 and above in 2022. This excludes admissions related to obstetrics and transfers from external institutions.^27^

#### Acute Conditions

PQI composite of acute conditions per 100,000 population, ages 18 years and older. Includes admissions with a principal diagnosis of one of the following conditions: dehydration, bacterial pneumonia, or urinary tract infection in 2022.^28^

#### Chronic Conditions

PQI composite of chronic conditions per 100,000 individuals aged 18 and above (i.e., diabetes complications, chronic obstructive pulmonary disease, asthma, hypertension, heart failure, or angina without a cardiac procedure) in 2022.^29^

#### Overall Conditions

PQI composite per 100,000 population, ages 18 years and older of all acute and chronic conditions in 2022.^30^

### Statistical Analysis

We analyzed the disease burden and quality of care across Ohio counties, as well as compared Appalachian (*n* = 32) and non-Appalachian (*n* = 56) regions in the state. Appalachian counties were classified based on the definitions set by the Appalachian Regional Commission.^31^ We compared the mean number of primary care physicians, specialty physicians, advanced practitioners, and other healthcare providers for Ohio counties using an ANOVA test. We broke down the mean prevalence, principal cost, and prevention quality indicator (PQI) by health condition. Next, we analyzed the effect of healthcare provider density (PCP, specialty physicians, advanced practitioners, and other healthcare providers) per 100,000 people on our primary outcome variable: the PQI across counties. Models were analyzed using generalized linear models for non-normal distributions. Likelihood ratio tests compared the fitted models to a null model in order to test overall model significance.

We examined differences in healthcare providers, disease burden, and quality of care between Appalachian and non-Appalachian regions. We calculated a proportions test to compare the percentage of counties that did not have various healthcare providers (PCP, specialty physicians, advanced practitioners, and other healthcare providers). Moreover, multiple *t*-tests compared the difference in the average rate of healthcare providers, prevalence of conditions, and prevention quality indicators.

The significance level for all tests in this study was set at *α* = 0.05. Models were compared using the Bayesian Information Criterion (BIC). The lowest BIC indicated the optimum model. A ΔBIC > 2 indicated significance, ΔBIC between 2 and 10 indicated moderate evidence, and ΔBIC > 10 indicated strong evidence favoring the model with a lower BIC.^32^ The collected variables and data were merged and analyzed using R-Studio, version 2023.03.1 + 446 (statistical computing program).

## RESULTS

This study looked at 15,413 (39.3%) specialty physicians, 9058 (23.1%) primary care physicians, 8345 (21.3%) other healthcare providers, and 6351 (16.2%) advanced practitioners (see Table 1). The mean number of providers per 100,000 people significantly differed between these four categories (*F* = 13.9, *P* < 0.001). The highest category was specialty physicians, which had a mean rate of 17.8 (SD = 14.2) providers per 100,000 people.

**Table 1:** Comparison of the Mean Number of Primary Care Physicians, Specialty Physicians, Advanced Practice Providers, and Other Healthcare Providers for Ohio Counties

	Mean provider rate (SD)*	***F* statistic**	***P*-value**
Primary care physicians	13.1 (8.0)	13.9	< 0.001
Specialty physicians	17.8 (14.2)		
Advanced practice providers	8.6 (7.5)		
Other healthcare providers	14.1 (6.4)		

^*^Mean provider rate was analyzed using ANOVA test

The mean prevalence and associated cost for various health conditions were calculated (see Table 2). The prevalence of hypertension (mean = 67.0, SD = 2.2) and diabetes (mean = 27.1, SD = 2.4) was the highest of the selected conditions. However, the highest average cost of the selected conditions was acute myocardial infarction (mean = 9663.3, SD = 2572.4) and COPD (mean = 1071.9, SD = 231.9).

**Table 2:** The Mean Prevalence and Cost for Various Health Conditions for Ohio Counties

Health conditions		
	Mean prevalence (SD)	Mean cost (SD)
Asthma	6.3 (1.0)	785.5 (253.4)
Acute myocardial infarction	1.3 (0.6)	9663.3 (2572.4)
COPD	15.0 (2.9)	1071.9 (231.9)
Diabetes	27.1 (2.4)	890.9 (146.4)
Hypertension	67.0 (2.2)	492.6 (96.6)
Preventable hospital stays	3140.5 (786.3)	-

The mean PQI was broken down by health condition (see Table 3). Short-term diabetes had the lowest average PQI across counties with a mean of 59.2 (SD = 48.9). This was significantly less than the PQI for long-term diabetes care (*P* < 0.001), which had a mean of 203.4 (SD = 92.1). Moreover, the number of preventable hospitalizations from chronic conditions (mean = 2024.9, SD = 526.8) was significantly higher (*P* < 0.001) than the number of preventable hospitalizations from acute conditions (mean = 851.6, SD = 262.2).

**Table 3:** The Mean Prevention Quality Indicator for Various Health Conditions for Ohio Counties

Prevention quality indicator (PQI)	
	Mean score (SD)
Asthma and COPD	351.6 (160.6)
Diabetes short-term complications	59.2 (48.9)
Diabetes long-term complications	203.4 (92.1)
Hypertension	105.2 (70.4)
Acute conditions	851.6 (262.2)
Chronic conditions	2024.9 (526.8)
Overall	2876.4 (688.9)

The population-adjusted rate of healthcare providers (PCP, specialty physicians, advanced practitioners, and other healthcare providers) per 100,000 people predicted the corresponding PQI across counties (see Table 4). Each healthcare provider category was significant in predicting the number of preventable hospitalizations for all the health conditions selected (*P* < 0.001). In addition, the multivariate mixed effects model of PCP*Specialist*APP*Other was also a significant predictor for all health conditions (*P* < 0.001).

**Table 4:** Modeling the Effects of Healthcare Providers on the Prevention Quality Indicator for Acute and Chronic Health Conditions

Model*	**Correlation coefficient (*r*)**	**BIC**	***P*-value**
**Asthma and COPD**			
PCP	0.7	172,434	< 0.001
Specialist	0.6	189,527	< 0.001
Advanced practice	0.6	194,411	< 0.001
Other	0.8	110,111	< 0.001
PCP*Specialist*APP*Other	1.0	15,765	< 0.001
**Diabetes short-term complications**			
PCP	0.7	19,636	< 0.001
Specialist	0.6	21,671	< 0.001
Advanced practice	0.6	21,819	< 0.001
Other	0.8	13,637	< 0.001
PCP*Specialist*APP*Other	0.9	5229	< 0.001
**Diabetes long-term complications**			
PCP	0.7	86,817	< 0.001
Specialist	0.6	95,383	< 0.001
Advanced practice	0.6	98,185	< 0.001
Other	0.8	54,818	< 0.001
PCP*Specialist*APP*Other	1.0	8065	< 0.001
**Hypertension**			
PCP	0.7	37,134	< 0.001
Specialist	0.7	40,182	< 0.001
Advanced practice	0.7	40,082	< 0.001
Other	0.8	23,506	< 0.001
PCP*Specialist*APP*Other	1.0	6218	< 0.001
**Acute conditions**			
PCP	0.6	508,547	< 0.001
Specialist	0.6	566,515	< 0.001
Advanced practice	0.6	571,755	< 0.001
Other	0.8	319,774	< 0.001
PCP*Specialist*APP*Other	1.0	37,155	< 0.001
**Chronic conditions**			
PCP	0.6	1,393,627	< 0.001
Specialist	0.6	1,506,845	< 0.001
Advanced practice	0.6	1,504,856	< 0.001
Other	0.8	862,792	< 0.001
PCP*Specialist*APP*Other	1.0	109,260	< 0.001
**Overall**			
PCP	0.6	2,112,850	< 0.001
Specialist	0.6	2,294,426	< 0.001
Advanced practice	0.6	2,292,983	< 0.001
Other	0.8	1,316,376	< 0.001
PCP*Specialist*APP*Other	1.0	158,538	< 0.001

^*^The models were analyzed using generalized linear models. The likelihood ratio test and BIC compared statistical significance and model fit

The best predictive model between PCP, specialty physicians, advanced practitioners, and other healthcare providers was other healthcare providers. There was strong evidence that other healthcare providers had a significant difference in BIC values for the PQI of asthma and COPD, short-term diabetes, long-term diabetes, hypertension, acute conditions, chronic, and overall conditions (ΔBIC > 10). The multivariate mixed effects model of PCP*Specialist*APP*Other significantly enhanced models predicting the number of preventable hospitalizations for short-term diabetes, long-term diabetes, hypertension, acute conditions, chronic, and overall conditions (ΔBIC > 10).

The differences in healthcare providers, disease burden, and prevention quality between Appalachian and non-Appalachian regions were depicted (see Table 5). There was a significant difference in the proportion of counties without specialist physicians (*P* < 0.05). Appalachia had 5/32 (15.6%) counties without a specialist physician or advanced practitioners. Non-Appalachia, however, had specialist physicians, primary care physicians, and other healthcare providers in every county. Moreover, the mean rate of other healthcare providers in Appalachian counties (mean = 10.9, SD = 5.7) was significantly less (*P* < 0.01) than the rate of other healthcare providers in non-Appalachian counties (mean = 15.9, SD = 6.1).

**Table 5:** Comparison of Healthcare Providers, Disease Burden, and Prevention Quality Between Appalachian and Non-Appalachian Regions

**Comparison of Appalachian and non-Appalachian regions**			
	**Appalachian (%)**	**Non-Appalachian (%)**	***P*-value**
**Number of counties without healthcare providers**			
PCP	2 (6.6%)	0 (0%)	0.3
Specialist physician	5 (15.6%)	0 (0%)	< 0.05
Advanced practice providers	5 (15.6%)	2 (3.6%)	0.1
Other healthcare providers	0 (0%)	0 (0%)	1.0
	**Appalachian mean (SD)**	**Non-Appalachian mean (SD)**	***P*-value**
**Healthcare providers**			
PCP rate (SD)	12.4 (10.2)	13.5 (6.4)	0.6
Specialist physician rate	14.7 (13.9)	19.6 (14.2)	0.1
Advanced practice providers	7.2 (7.8)	9.4 (7.3)	0.2
Other healthcare providers	10.9 (5.7)	15.9 (6.1)	< 0.01
**Prevalence of health conditions**			
Asthma	6.3 (1.1)	6.3 (1.0)	0.9
Acute myocardial infarction	1.7 (0.7)	1.2 (0.4)	< 0.001
COPD	17.4 (2.8)	13.7 (1.9)	< 0.001
Diabetes	28.4 (2.3)	26.3 (2.1)	< 0.001
Hypertension	67.6 (2.3)	66.7 (2.1)	0.1
**Prevention quality indicator (PQI)**			
Asthma and COPD	359.6 (210.1)	347.0 (125.9)	0.8
Diabetes short-term complications	69.4 (62.4)	53.4 (38.8)	0.2
Diabetes long-term complications	197.5 (119.3)	206.8 (73.3)	0.7
Hypertension	102.1 (83.2)	107.0 (62.7)	0.8
Acute conditions	891.5 (324.3)	828.9 (219.1)	0.3
Chronic conditions	2140.1 (690.5)	1959.0 (397.5)	0.2
Overall	3031.5 (883.7)	2787.8 (537.2)	0.2
Preventable hospital stays	3237.8 (1013.3)	3084.9 (624.9)	0.4

Note: Number of counties without healthcare providers was compared using proportion tests

Note: Mean rates were compared using *t*-tests

Appalachian counties had a significantly higher prevalence of acute myocardial infarction, COPD, and diabetes (*P* < 0.001). Specifically, COPD (mean = 17.4, SD = 2.8) and diabetes (mean = 28.4, SD = 2.3) in Appalachian counties were significantly higher (*P* < 0.001) than COPD (mean = 13.7, SD = 1.9) and diabetes (mean = 26.2, SD = 2.1) in non-Appalachian counties.

## DISCUSSION

The objective of this study was to assess disease burden and examine associations with the number of healthcare providers in Ohio counties, with a focus on Appalachian versus non-Appalachian regions. This study aimed to identify whether the prevalence of providers impacted the PQI of various conditions.

We found that the PQI was higher for chronic conditions compared to acute conditions, indicating the need for improved outpatient care to avoid preventable hospital admissions for chronic conditions.

Furthermore, Appalachian counties had higher prevalence rates of acute myocardial infarction, COPD, and diabetes. Contrary to our hypothesis, no significant differences in PQI were observed between Appalachian and non-Appalachian counties for asthma, acute myocardial infection, hypertension, COPD, and diabetes. The PQI metric reflects the number of preventable hospitalizations with adequate outpatient care. Our findings suggest that while both regions may face similar challenges in managing these conditions, the prevalence rate varies between Appalachian and non-Appalachian counties.

When analyzed by county, there was a positive correlation between PQI and the rate of healthcare providers. At first glance, this correlation might suggest non-efficacious outpatient care, but a higher concentration of healthcare providers does not necessarily mean lower quality of care. The PQI is gathered from discharge data of patients who have been hospitalized due to preventable circumstances. Nonetheless, many patients in counties with a low prevalence of providers must either seek out or be transported to counties with resources to treat them. Larger hospitals and higher-level trauma centers traditionally have more resources and providers to treat patients with acute and chronic conditions in an emergency setting.^33^ A county with a high concentration of providers within its hospital network may attract more patients whose outpatient care is conducted in a different county with fewer providers.

Another explanation for this correlation could be a lack of access or confidence in the healthcare received. As previously mentioned, people in Appalachian Ohio are more likely to report having inadequate healthcare services despite no significant difference in PCP rate.^34^ The same study also showed significant dissatisfaction with the care available to Appalachian Ohioans concerning convenience, quality, and information provided.^34^ It is important to highlight the perspectives of the individuals from underserved communities that studies like this aim to shed light on and garner support for. This could be a productive avenue for future research to further explore the gaps in healthcare and the causes of disparities as perceived by these individuals. Alternative measures for quality of care, such as patient-reported outcomes or mortality rates, could provide a more comprehensive assessment of healthcare efficacy.

Appalachian regions face many disparities in healthcare, such as increased rates of diabetes, cancer, and depression and lower levels of perceived availability and satisfaction with the services available. These disparities likely contribute to ongoing health inequities, necessitating targeted healthcare and policy interventions that increase provider accessibility and patient education.^22,24^ A previous study found that individuals in these regions self-reported a lack of healthcare services in their county when compared to perceptions in non-Appalachian counties.^34^ In contrast, the current study found that the actual difference in primary care provider rate between Appalachian and non-Appalachian regions was lower but not significant. Although the difference in primary care provider rate was not statistically significant, this finding should not undermine patients reported difficulty in access to care.^34^ Patients in rural areas, while having a similar patient to PCP ratio, have been shown to have further travel times to access providers.^8,9,22^ Factors such as transportation barriers and travel time can significantly reduce the accessibility and efficacy of healthcare in rural areas.^8,9^

We observed a significant difference in the mean prevalence of other healthcare providers. In addition, the proportion of counties that did not have a specialist physician was 5/32 (15.6%) in Appalachian counties versus 0/56 (0%) in non-Appalachian counties (Table 5). The lack of providers may pose a barrier to healthcare access, such as workforce shortages, increased travel time and financial burden, delayed diagnoses and treatments, and disrupted continuity of care.^7–9^

The need for “other healthcare providers” in Appalachian counties exposes gaps in collaborative efforts to manage patients holistically. For example, patients with type 2 diabetes who are referred to a nutritionist or dietitian have a substantial reduction in their hemoglobin A1C.^35^ In instances where these alternative providers are not readily available, PCPs may find themselves shouldering extra responsibilities without the ability to enlist collaborators or delegate certain aspects of patient care.

The rate of cardiovascular physicians (MD) in Franklin County near Columbus, Ohio, is 10.59 per 100,000.^36^ In contrast, several of the Appalachian counties in Ohio have a rate of zero cardiovascular physicians (MD).^36^ This suggests that Appalachian counties may experience barriers to care in urgent health issues such as acute myocardial infarction, where time is an important factor in outcomes.

In terms of the efficacy of preventive care for chronic versus acute conditions in Ohio, chronic conditions tended to have higher PQIs. A higher PQI describes increased incidences of preventable hospitalizations, which can be demonstrative of a lack of access to efficacious outpatient care and support.^25^ The debilitation, pain, uncertainty, and complex treatment regimens associated with chronic illness can culminate in clinical burnout for the patient and their caretakers.^37^ Clinical burnout is characterized by emotional/physical exhaustion, apathy, and counterproductive behaviors, such as avoiding medical appointments or delaying care.^37,38^ This may explain why chronic illnesses lag behind acute conditions in terms of preventable hospitalizations.^37^

It is worth considering how increasing the availability and ease of access to supportive resources could prevent these hospitalizations. Patients who have logistical barriers to care for their chronic condition may increase their risk of potential complications.^7–9^ Given the higher preventable mortality rate in Ohio relative to the national average (95.9 vs 84.5), attacking the issue of inadequate preventive outpatient care for asthma, COPD, and other chronic illnesses should be prioritized.^5^

Appalachian regions in Ohio experience higher rates of COPD, diabetes, and acute myocardial infarction compared to non-Appalachian regions. This could motivate policymakers and healthcare systems to focus efforts on improving Appalachian health inequities. It is important to note that while hypertension and diabetes have the highest prevalence across the state, acute myocardial infarction and COPD, respectively, are the costliest of the medical conditions included in this study. Hypertension and diabetes increase the likelihood of developing more severe and costlier complications, such as acute myocardial infarction and COPD. These conditions are preventable, and it is relevant to recognize that the lower number of PCPs in Appalachia, although not statistically significant, could be an important factor contributing to a lack of prevention education in this patient population.

Future studies could explore the barriers as perceived by Appalachian residents, which could guide more effective health interventions and treatments. Additional future research could analyze mortality rates and how they correlate with the number of physicians in the context of Appalachian vs non-Appalachian counties. It would be beneficial to assess if the reduced number of physicians/specialists in Appalachia correlates with higher mortality and morbidity rates. Such analysis could be important in advocating for more healthcare providers to practice in rural regions, ensuring a higher standard of care and greater opportunities for preventive education.

### Limitations

Limitations to this study include the following: data completeness and accuracy, generalizability of the data, potential confounding variables, lack of clinical data, and the cross-sectional study design. First, CMS data were vulnerable to incompleteness, coding errors, and delays in reporting. Next, the data are specific to Medicare and Medicaid beneficiaries and thus may not be generalizable to the general population. Additionally, this data does not include important confounding variables, such as lifestyle factors, social determinants of health, and detailed clinical information (e.g., laboratory results, imaging findings). Also, counties can be diverse and vary in population density, socioeconomic status, and healthcare access. As such, county-level data aggregates information, potentially masking important variability and perceptions of individuals within the county. Finally, the cross-sectional nature of this study restricted the interpretation of causal relationships between variables.

To address potential limitations in our study, we adjusted our statistical models to account for confounding variables, such as population density. The statistical analysis also implemented generalized linear models to account for non-normal distributions. We used county-level data from CMS, which is the single largest payer for healthcare services in the United States. This was to ensure a comprehensive and broad overview of the state of Ohio and regions within.

## CONCLUSION

In Ohio, non-physician healthcare providers were more prevalent than PCPs and specialist physicians. Hypertension and diabetes were the most prevalent health conditions, while acute myocardial infarction and COPD were the costliest. This study found higher PQI scores for chronic conditions, indicating the need for higher-quality outpatient care to prevent avoidable hospital admissions. A higher number of all four provider types correlated with a higher PQI for all conditions measured in this study. Finally, Appalachian counties had fewer other healthcare providers and many counties lacked specialist physicians, highlighting significant disparities in healthcare access in Appalachian Ohio.

## Data Availability

The dataset generated and analyzed during the current study is available from the Centers for Medicare & Medicaid Services (CMS).
